# A Different Type of Tennis Elbow: Spontaneous Septic Arthritis of the Elbow in a Previously Healthy 40-Year-Old Male

**DOI:** 10.7759/cureus.87228

**Published:** 2025-07-03

**Authors:** Miguel F Agrait Gonzalez, Adriana Mercado, Samuel Ortiz Diaz

**Affiliations:** 1 Emergency Medicine, Hospital San Lucas Episcopal, Ponce, PRI; 2 Emergency and Sports Medicine, School of Medicine, West Virginia University, Morgantown, USA; 3 Emergency Medicine, Centro Médico Episcopal San Lucas, Ponce, PRI

**Keywords:** arthrocentesis, elbow effusion, elbow infection, elbow ultrasound, msk ultrasound, point-of-care-ultrasound, pyogenic arthritis, septic arthritis

## Abstract

Septic arthritis of the elbow is a serious and uncommon condition, particularly in immunocompetent individuals and those without risk factors. We present the case of a 40-year-old previously healthy male who developed spontaneous elbow septic arthritis after initiating a recreational tennis regimen. He presented with progressive elbow pain, loss of extension, and swelling, but lacked fever or any systemic findings suggestive of infection on arrival. Point-of-care ultrasound (US) revealed an elbow effusion, which then prompted US-guided arthrocentesis in the emergency department. Synovial fluid analysis demonstrated 57,000 white blood cells with 99% polymorphonuclear cells. Additionally, the Gram stain was positive for Gram-positive cocci in clusters, confirming the presence of intraarticular infection. The patient was admitted for surgical joint washout and IV antibiotics. He was able to complete a course of IV and then oral antibiotics and had a full functional recovery at six months. This case highlights the utility of bedside US in recognizing joint effusions and guiding arthrocentesis, even in patients without typical risk factors or signs and symptoms of systemic illness.

## Introduction

Septic arthritis is a rapidly progressive joint infection that requires urgent recognition and intervention to prevent permanent joint damage, systemic spread, significant morbidity, and mortality. It is estimated to occur in 4-10 per 100,000 person-years in the general population and up to 70 per 100,000 in those with risk factors such as rheumatoid arthritis or immunosuppression [1,2]. The knee is the most commonly affected joint, followed by the hip, shoulder, and elbow [3]. Elbow involvement accounts for only 9% to 10% of native joint infections [4], making it an uncommon but still potentially important cause of articular pain in adults.

Most patients with septic arthritis will have at least one risk factor such as diabetes mellitus, intravenous drug use, HIV infection, preexisting joint disease, recent joint surgery, previous joint injections, overlying skin infection, or other systemic infection [5,6]. However, 15% of all septic arthritis cases occur in otherwise healthy individuals without an identifiable predisposing factor [7]. In these patients, diagnosis is frequently delayed, as symptoms are often mistaken for more benign musculoskeletal (MSK) conditions such as lateral epicondylopathy, olecranon bursitis, soft tissue strain, or other similar overuse syndromes. Unfortunately, joint destruction can begin in as little as 24-48 hours from infection onset, emphasizing the importance of early detection and management [1].

Physical exam findings such as joint swelling, warmth, and pain are common but nonspecific. An inability to extend the elbow fully when compared to the unaffected extremity should raise suspicion for an intraarticular process leading to effusion, such as gout, fracture, degenerative arthritis, or septic arthritis. External (i.e., non-articular) pathologies such as olecranon bursitis, medial, and lateral epicondylopathy do not result in loss of extension to passive and active motion, although the pain and swelling may be similar in presentation in certain cases [8]. Fractures that may be missed or occult on X-ray may only present with the finding of joint effusion. In recent years, MSK ultrasound (US), especially when used at the point-of-care US (POCUS), has emerged as an important tool in the early evaluation of joint pain. Bedside US is highly sensitive for detecting effusions, including small-volume fluid collections that may not be appreciated on physical exam or plain radiographs [9]. It also allows for safe and accurate image-guided arthrocentesis, expediting diagnostic confirmation and appropriate management [10].

In this report, we describe a case of spontaneous septic arthritis of the elbow in a healthy adult male with no underlying risk factors. We highlight the key clinical clues that led to the diagnosis and emphasize the utility of POCUS in the early recognition and management of joint effusions and infections in the emergency setting.

This article was previously presented as a poster at the American Medical Society for Sports Medicine Annual Conference on April 14, 2024, in Baltimore, MD.

## Case presentation

A 40-year-old previously healthy male presented to the emergency department (ED) with a one-week history of right elbow swelling and progressively worsening pain. Symptoms began one week after starting a daily recreational tennis program, which included at least 2 hours of daily play. He reported a limited range of motion, particularly in extension, but denied any trauma, fever, chills, systemic symptoms, or history of prior joint problems. His medical history was unremarkable without any history of diabetes, immunosuppression, intravenous drug use, arthritis, or recent infections. He had no prior joint surgeries or injections. He denied any sexual contact outside of his long-term female partner. The patient had a delayed presentation as he had been told by his trainer that he was dealing with *tennis elbow* and was provided with rehab and strengthening exercises, which did not improve the issues with range of motion or pain.

**Figure 1:**
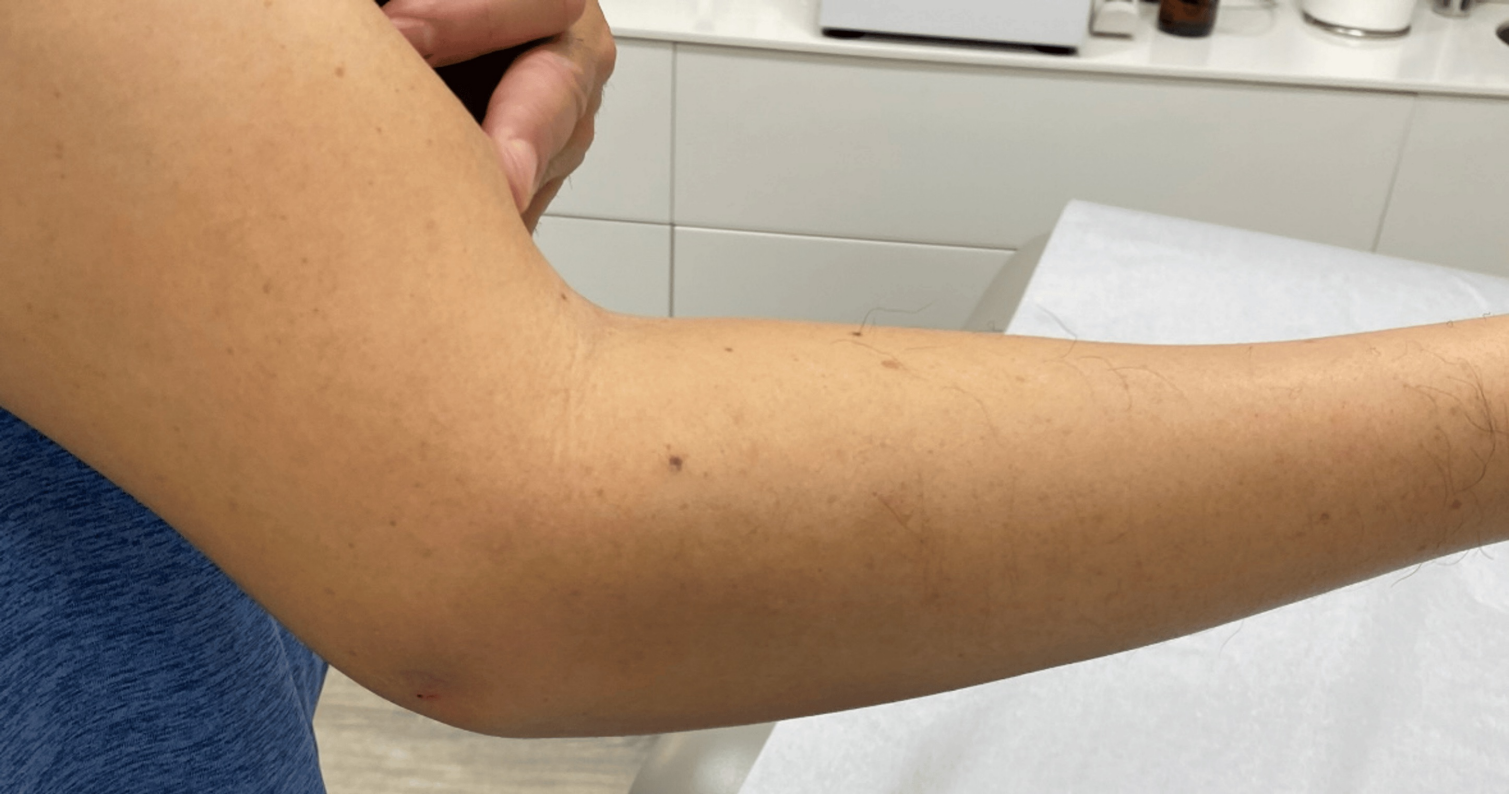
Patient's arm held in full extension on presentation. Note the lack of obvious swelling, erythema, or other significant skin findings to suggest infection. Image obtained and used with the patient's consent.

On examination, the patient held his right arm in partial flexion. Active extension was limited due to pain, and there was also a limitation on passive extension as the elbow did not extend beyond a certain range (Figure 1).

The elbow was diffusely tender throughout the anterior joint line, triceps tendon, olecranon, and common extensor tendon/lateral epicondyle area. There was warmth appreciated on palpation, but without erythema or skin changes. Vitals were within normal limits, with no fever or tachycardia. 

**Figure 2:**
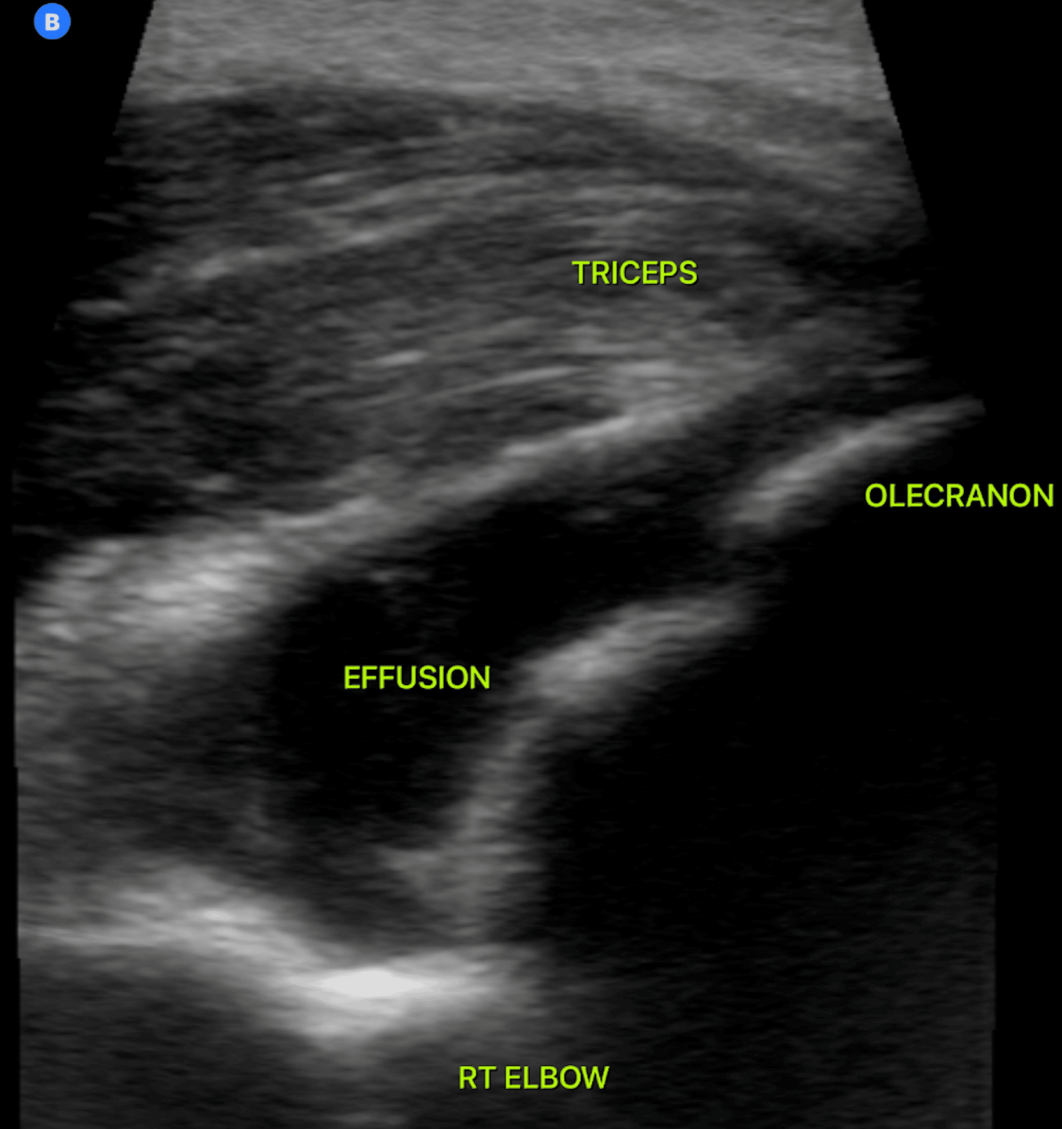
Ultrasound image of the patient’s right elbow. The probe is positioned longitudinally over the triceps tendon, with the tip placed on the olecranon. Any fluid visualized inferior to the triceps tendon is located intra-articularly.

Point-of-care MSK US demonstrated an intraarticular effusion (Figure 2).

**Figure 3:**
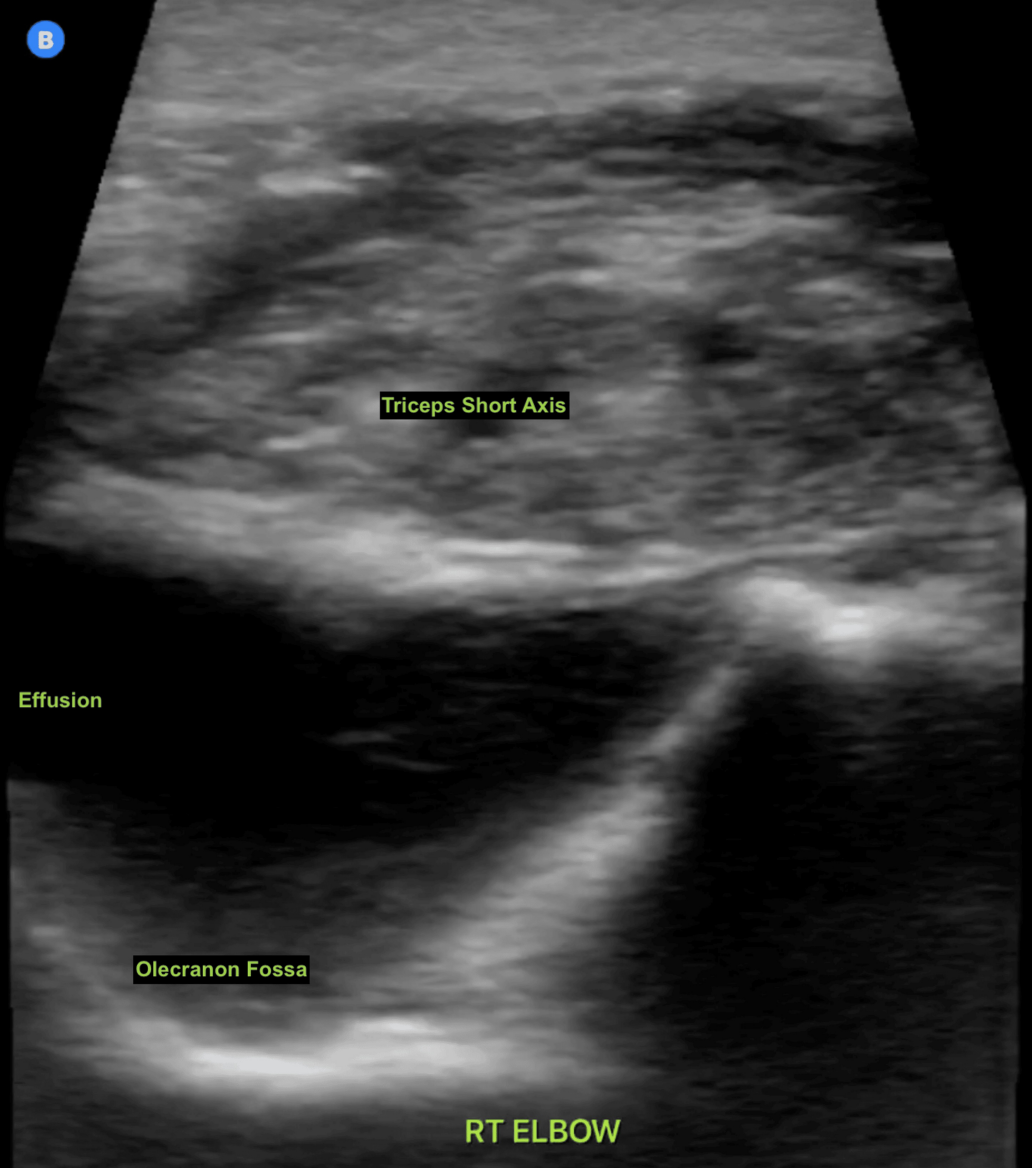
Transverse (short-axis) view of the right elbow joint showing an intra-articular effusion. This view allows in-plane needle guidance into the joint for arthrocentesis.

**Figure 4:**
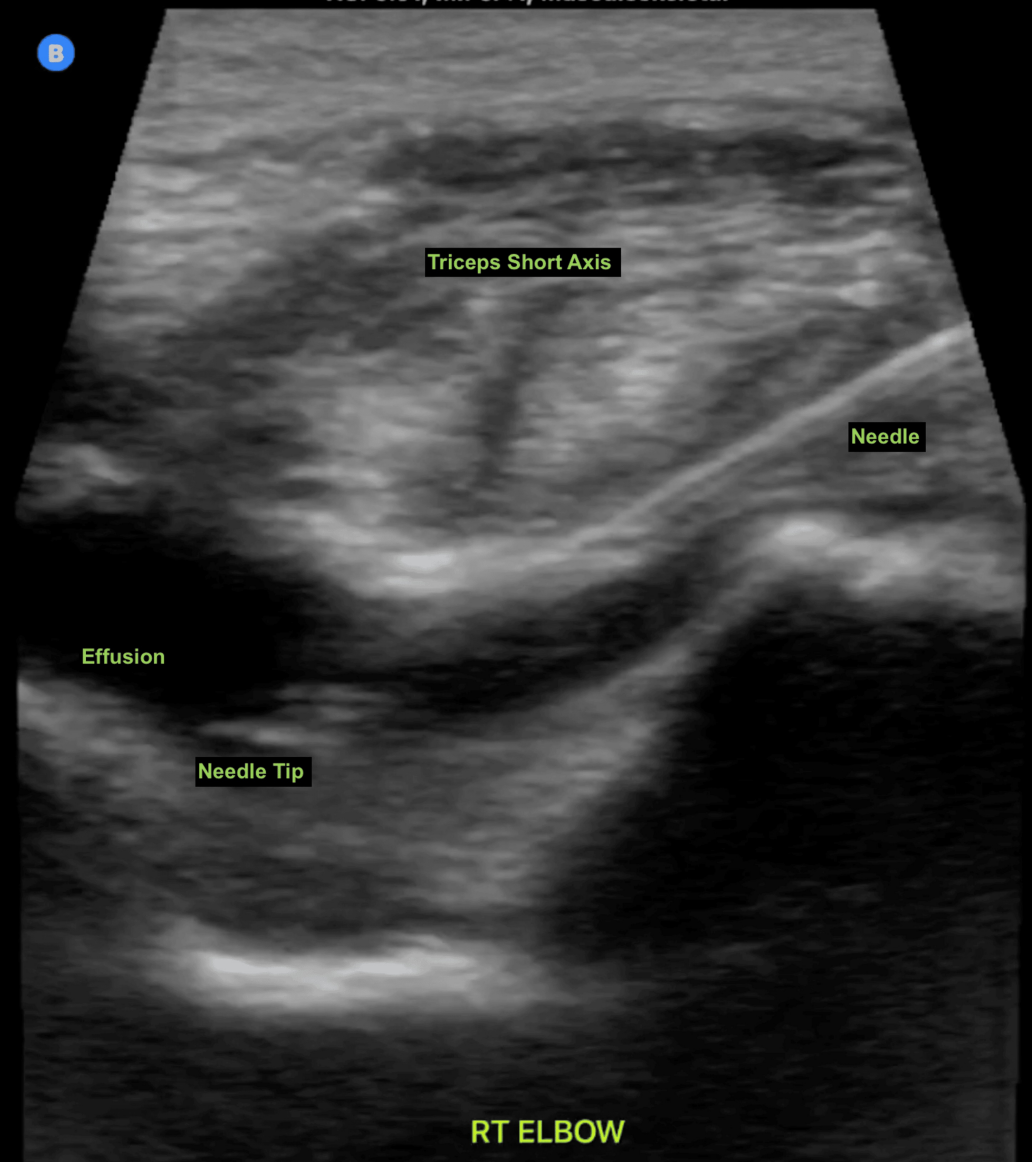
POCUS-guided elbow arthrocentesis. The needle is visualized in long axis, with the tip located within the joint effusion. POCUS, point-of-care ultrasound

In the presence of an unexplained and atraumatic effusion, the decision was made to further evaluate with US-guided arthrocentesis and synovial fluid analysis to exclude a potential septic joint (Figures 3, 4).

Laboratory tests in the ED revealed a synovial white blood cell (WBC) count of 57,000/mm³ with 99% polymorphonuclear cells. Gram stain was reported shortly thereafter and showed Gram-positive cocci in clusters. Crystals were absent. Synovial fluid analysis is consistent with septic arthritis, which is generally diagnosed when the WBC count exceeds 50,000 or the polymorphonuclear cell (PMN) percentage is greater than 90%. Bloodwork was notable for mild leukocytosis, along with elevated C-reactive protein (CRP) and erythrocyte sedimentation rate (ESR) (Tables 1, 2). 

**Table 1:** Synovial fluid and whole blood results with reference ranges. WBC, white blood cell; PMN, polymorphonuclear cell

	Synovial results	Reference values
Color	Yellow/green	Clear
Appearance	Purulent	Transparent
WBC count	57,000 cells/mm^3^	<200 cells/mm^3^
PMN (%)	99%	<25%
Gram stain	Gram + cocci in clusters	No bacteria seen
Crystals	No crystals seen	No crystals seen

**Table 2:** Blood test results with reference ranges. WBC, white blood cell; CRP, C-reactive protein

	Blood	Reference values
WBC count	13,100 WBC/μL	4,500-11,000 WBC/μL
Hemoglobin	14.8 g/dL	14-18 g/dL
Hematocrit	44%	42%-52%
Platelet count	373,500	150,000-450,000/μL
CRP	14.6 mg/dL	<2.0 mg/dL
Sedimentation rate	46 mm/hour	<20 mm/hour
Lactic acid	1.7 mmol/L	<2.0 mmol/L

Blood cultures were drawn, and the patient was started empirically on intravenous vancomycin and ceftriaxone.

He was admitted to the orthopedic service and taken to the operating room on the day of his ED presentation for irrigation and debridement of the elbow joint. Operative findings included gross purulence within the joint. Synovial fluid cultures returned positive for methicillin-sensitive Staphylococcus aureus (MSSA) 3 days later (Table 3).

**Table 3:** Antibiotic susceptibility panel of the patient’s synovial fluid sample showed pan-sensitive Staphylococcus aureus (MSSA). MIC, minimum inhibitory concentration

Antibiotic susceptibility panel	Organism identified: Staphylococcus aureus	
Antibiotic	MIC value (μg/mL)	Interpretation
Amoxicillin/Clavulanate	≤2/1	Sensitive
Ampicillin/Sulbactam	≤8/4	Sensitive
Ampicillin	≤0.25	Sensitive
Azithromycin	≤2	Sensitive
Cefazolin	≤1	Sensitive
Ceftriaxone	≤1	Sensitive
Ciprofloxacin	≤2	Sensitive
Clindamycin	≤0.5	Sensitive
Daptomycin	≤1	Sensitive
Erythromycin	≤0.5	Sensitive
Gentamicin	≤1	Sensitive
Levofloxacin	≤1	Sensitive
Linezolid	≤2	Sensitive
Meropenem	≤2	Sensitive
Oxacillin	≤0.25	Sensitive
Penicillin	≤0.12	Sensitive
Rifampin	≤1	Sensitive
Tetracycline	≤1	Sensitive
Trimethoprim/Sulfamethoxazole	≤1/19	Sensitive
Vancomycin	≤1	Sensitive

Antibiotic coverage was then narrowed to intravenous cefazolin once culture results became available.

The patient recovered well postoperatively and was discharged on post-op day 7 with two additional weeks of home IV cefazolin, and then transitioned to oral amoxicillin to complete a 42-day course of antibiotics, as per infectious disease recommendations. At six-month follow-up, he had a full return to baseline activity and no residual joint dysfunction.

## Discussion

Septic arthritis of the elbow in an immunocompetent adult is rare but clinically significant. While the elbow is involved in less than 10% of septic arthritis cases [4], its complex structure and limited joint space predispose it to rapid compromise once infected. This case illustrates that even in the absence of systemic illness or identifiable risk factors, clinicians must consider joint infection in the differential diagnosis of atraumatic joint effusion, especially as there are no sufficiently sensitive or specific physical examination or laboratory findings to exclude a septic joint without synovial fluid analysis [11]. Any unexplained effusion should at least prompt consideration of arthrocentesis to evaluate possible etiologies and exclude infection as the source of the effusion. 

A key clinical finding in this case was progressive loss of elbow extension. This subtle sign should raise concern for an intraarticular process, particularly when accompanied by swelling and warmth [8]. In such cases, POCUS offers a rapid, noninvasive, and highly sensitive tool to detect joint effusion and potentially guide diagnostic aspiration. In a study of elbow joint pathology, bedside US had a near 100% sensitivity for detecting effusion compared to physical examination [9]. It is particularly useful in irregular joints like the elbow, where effusions can be difficult to appreciate on exam alone. Evaluating for effusions using MSK POCUS should be considered a core skill for emergency physicians who evaluate joint complaints regularly. Elbow POCUS for effusion in particular requires only a single view, making it a simple skill to obtain and train for regular use in the ED.

US guidance for arthrocentesis, as opposed to traditional palpation-guided techniques, improves procedural success dramatically and can accelerate time to diagnosis, allowing for early initiation of antibiotics and surgical consultation [12]. In the ED setting, POCUS can potentially reduce the time to diagnosis and intervention, as it did in this particular case.

This case reinforces that even a well-appearing patient with joint swelling and pain should not be dismissed. In the absence of trauma, early imaging and aspiration are warranted for any unexplained effusion. As demonstrated here, integration of MSK US into the ED workflow can facilitate the diagnosis of an effusion and the evaluation of said effusion using arthrocentesis if necessary. This, in turn, allows prompt management and more appropriate disposition.

## Conclusions

Spontaneous septic arthritis of the elbow is an uncommon presentation, particularly in healthy adults without identifiable risk factors. However, clinicians must maintain high suspicion when encountering atraumatic joint swelling with progressive limitation in range of motion, especially loss of terminal extension.

Point-of-care MSK US is a rapid, effective tool for identifying elbow effusions and guiding diagnostic aspiration. Its early use in the ED can significantly reduce diagnostic delay and improve clinical outcomes in patients with septic arthritis.
